# Adverse Events Following Administration of Anti-CTLA4 Antibody Ipilimumab

**DOI:** 10.3389/fonc.2021.624780

**Published:** 2021-03-09

**Authors:** Amirali Karimi, Sanam Alilou, Hamid Reza Mirzaei

**Affiliations:** ^1^ School of Medicine, Tehran University of Medical Sciences, Tehran, Iran; ^2^ Department of Medical Immunology, School of Medicine, Tehran University of Medical Sciences, Tehran, Iran

**Keywords:** CTLA4, Anti-CTLA4 antibody, ipilimumab, adverse events, cancer immunotherapy

## Abstract

Ipilimumab, a monoclonal anti-CTLA4 antibody, paved the path for promising treatments, particularly in advanced forms of numerous cancers like melanoma. By blockading CTLA-4, ipilimumab can abolish the higher binding affinity of B7 for CTLA-4, setting CD28 free to act unlimited. This blockade can result in an amplified antitumor immune response, and thereby, boosting more effective tumor regression. However, this blockage can lead to diminished self-tolerance and yielding autoimmune complications. The current review aims to describe adverse events (AEs) following the administration of ipilimumab in different cancers as every benefit comes at a cost. We will also discuss AEs in two different categories, melanoma and non-melanoma, owing to the possible shining promises in treating non-melanoma cancers. As the melanoma settings are more studied than other cancers, it might even help predict the patterns related to the other types of cancers. This similarity also might help physicians to predict adverse events and correctly manage them in non-melanoma cancers using the extensive findings reported in the more-studied melanoma settings. Recognizing the adverse events is vital since most of the adverse events could be reverted while carefully implementing guidelines. Finally, we will also describe the observed effectiveness of ipilimumab in non-melanoma cancers. This effectiveness reveals the importance of understanding the profile of adverse events in this group, even though some have not received FDA approval yet. Further clinical trials and careful systematic reviews may be required to decipher the hidden aspects of therapies with ipilimumab and its related AEs.

## Introduction

Following a tumor antigen presentation by major histocompatibility complex (MHC) class I or II expressed on the antigen-presenting cells (APCs) and a vital second costimulatory signal together can fully promote T cell activation. This signal is transduced through B7-1 (CD80) or B7-2 (CD86) on the APC cell surface upon ligation to CD28 on the T cell surface. CTLA-4 (or CD152), with its superior affinity for B7, could significantly bypass CD28 activation, promote T cell anergy and inhibit T cell activation and IL-2 production (1–5).

Ipilimumab, a monoclonal antibody (MAB) blockading CTLA-4, can abolish the higher binding affinity of B7 for CTLA-4, setting CD28 free to act unlimitedly. This blockade would result in an amplified immune stimulation, boosting tumor annihilation (6). Fortunately, many studies have found that ipilimumab is effective in the treatment of several disorders (7). However, it can be predicted that the blockage of B7: CTLA-4 interaction may come out with autoimmune complications, as CTLA-4 signaling plays a critical role in the maintenance of self-tolerance to self-antigens (6, 8, 9).

Ipilimumab increases absolute cell counts of eosinophil, lymphocytes, effector T cells, and their activation level. On the other hand, it also has a negative impact on some immunosuppressive components of the immune system such as myeloid derivative suppressor cells (MDSC) and regulatory T cells (10). Therefore, physicians should keep ipilimumab in mind as a possible cause of immune-related adverse events (irAEs). Interestingly, there might be a correlation between higher irAEs and better response to treatment (11, 12). The significance of such correlations is still a matter of debate (11, 13, 14)

**Figure 1:**
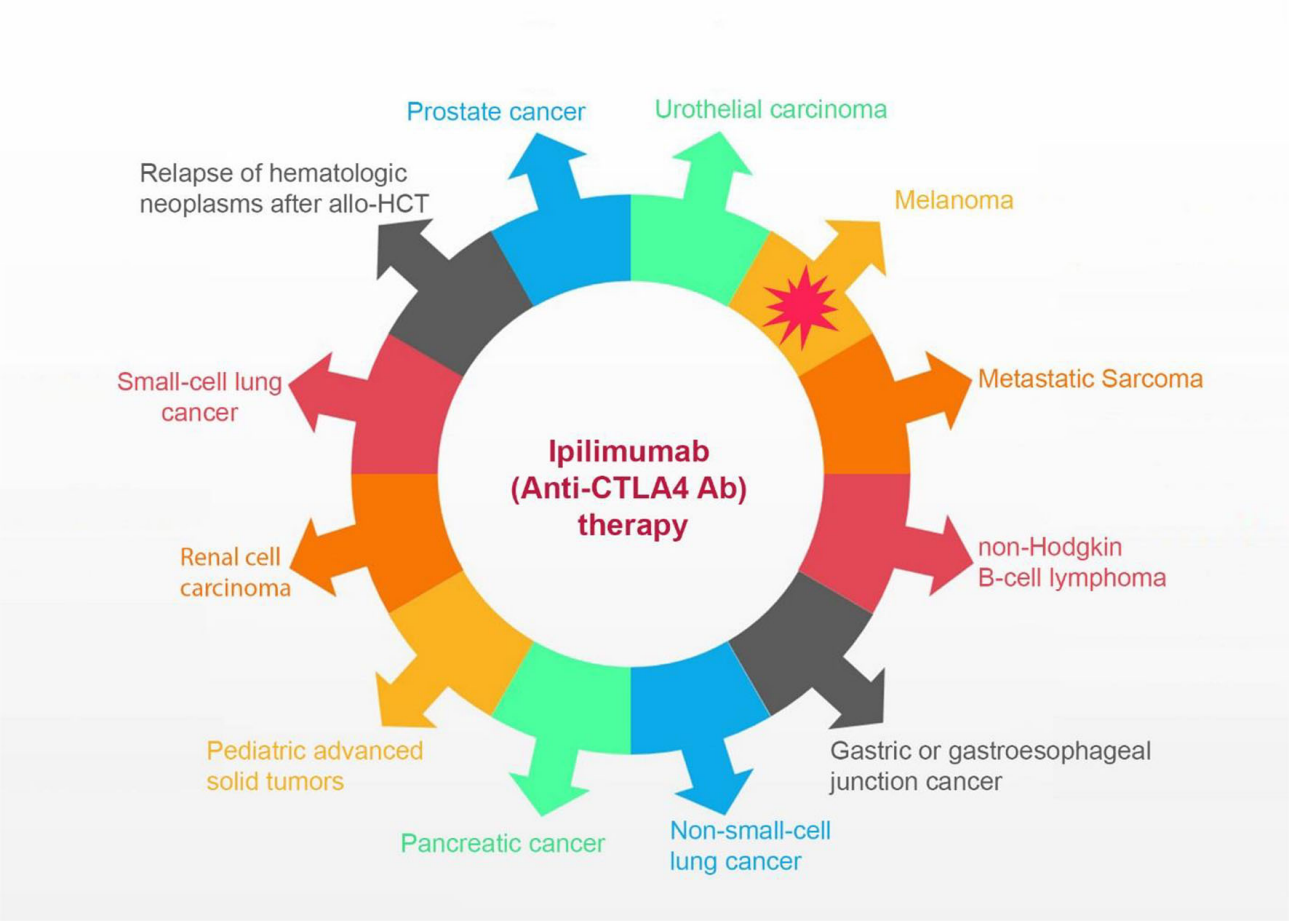
The spectrum of activity of ipilimumab in melanoma and non-melanoma cancers.

The current review aims to discuss adverse events (AEs) following the administration of ipilimumab in different cancers, as every benefit comes at a cost (**Figure 1**). We will discuss AEs in two different categories, melanoma and non-melanoma. As the melanoma setting is more studied than other cancers, it might even help physicians predict the patterns related to the other types of cancers. Furthermore, we will discuss the evidence-based effectiveness of ipilimumab in non-melanoma cancers. This effectiveness reveals the significance of AE profiling this group, even though some have not received FDA approval yet.

## Effectiveness and Adverse Events in Non-Melanoma Cancers

Some studies have shown that combined treatment with ipilimumab, carboplatin, and etoposide drugs as well as ipilimumab plus nivolumab could benefit patients with advanced small-cell lung cancer (SCLC) and recurrent SCLC patients respectively (15, 16). Conversely, two studies have not observed a significant increase in the overall survival or progression-free survival in the patients treated with ipilimumab plus carboplatin and paclitaxel (17), or etoposide and platinum (18) in extensive-disease-SCLC. This was also evident in patients with squamous non-small lung cancer (NSCLC) who were co-treated with ipilimumab and chemotherapeutic drugs (19). However, ipilimumab was found to be highly effective in combination with either nivolumab or carboplatin and paclitaxel in advanced NSCLC (20–22). The recommended dose of ipilimumab was 10 mg/kg combined with carboplatin and paclitaxel in Japanese patients with NSCLC (23). In stage II-IIIA NSCLC patients, neoadjuvant use of ipilimumab plus platinum doublet regimen chemotherapy found to be safe and achievable before surgical resection (24).

Ipilimumab was also increased progression-free survival, and prostate-specific antigen (PSA) rates were responded better in the ipilimumab group versus placebo in metastatic castration-resistant prostate cancer (mCRPC) (25–27). Still, they could not prolong overall survival in some studies (26, 27). Nevertheless, a dose-escalation clinical trial study has predicted a prolonged overall survival with prostate GVAX/ipilimumab, though its small study population and lack of a control group call its reliability into question (28). Slovin et al. have found a clinical antitumor response following administration of 10 mg/kg ipilimumab with or without radiotherapy in mCRPC (29). In metastatic urothelial cancer, although chemotherapy plus ipilimumab regime could not improve 1-year overall survival, the regime was feasible and could confer appropriate cytotoxic backbones (30). In a recent study, combination therapy with nivolumab plus ipilimumab could result in high antitumor activity with a manageable safety profile in patients with metastatic urothelial carcinoma (31). Like other types of cancer, treatment with ipilimumab plus nivolumab could improve the overall survival in patients diagnosed with metastatic renal cell carcinoma (mRCC) (32, 33). Ipilimumab monotherapy also resulted in some cancer regression cases in mRCC, even in cases with no response to prior immunotherapies (34). In an interesting study, ipilimumab monotherapy was compared to best supportive care (BSC) in metastatic or unresectable locally advanced forms of gastric or gastroesophageal junction tumors. It has been shown that, although ipilimumab monotherapy could not improve immune-related progression-free survival (irPFS), a comparable median overall survival of approximately 1 year and a satisfactory safety profile highlight the significance of the ipilimumab combination therapy in gastric cancer (35). Combination therapy with ipilimumab plus GM-CSF cell-based vaccines (GVAX) could also improve antitumor immune response in the previously treated advanced pancreatic ductal adenocarcinoma (PDA), paving the path for future studies in the field (36). However, ipilimumab alone seems to be ineffective in locally invasive or advanced pancreatic cancer, except for a patient with a significant response, rescuing hope for further attempts and approaches toward immunotherapy, in particular combination immunotherapies (37). Consistent with the above findings, it has been exhibited that single-agent ipilimumab therapy which was implemented on pediatric patients (21 years old or younger) with advanced solid tumors, showed increased survival in a group of patients experiencing irAEs compared to tolerant patients. However, no objective antitumor response was reported (38).

In certain types of sarcoma, ipilimumab plus nivolumab was also found to be promising (39). Ipilimumab has elicited antitumor activity in B-cell non-Hodgkin Lymphoma patients who had relapsed or refractory type of the disease (40). In the context of recurrence hematologic neoplasms following an allogeneic hematopoietic stem cell transplantation (allo-HCT), ipilimumab could be a choice. Still, immune-related complications and graft-versus-host disease (GVHD) occurred in the cases (41). Another study has also established the effectiveness of ipilimumab in the provocation of Graft-versus-malignancy (GVM) following allo-HCT, yet they found no graft rejection or GVHD induced by ipilimumab (42). On the other hand, a specific murine anti-CTLA-4 MAB has been useful in inhibiting chronic GVHD mediated by Th-2 in the mice (43). **Table 1** demonstrates a quick review of the non-melanoma clinical trials discussed earlier. By accurately taking prior studies into account and designing further studies, we may cover the drawbacks of ipilimumab monotherapy with the combinatorial therapeutic approaches. It will also help us compare studies and drug regimens and avoid imposing unwanted side effects on the patients.

**Table 1:** Selected studies evaluating ipilimumab in non-melanoma cancers.

Type of cancer	Study evaluating Ipilimumab monotherapy	Study evaluating Ipilimumab combination therapy	Study evaluating Ipilimumab both alone and combination therapy
**Prostate cancer**	Beer et al. (26) Kwon et al. (27)	Jochems et al. (25) Santegoets et al. (28)	Slovin et al. (29)
**Non-small-cell lung cancer**	N/A	Govindan et al. (19) Hellmann et al. (20) Hellmann et al. (21) Lynch et al. (22) Horinouchi et al. (23) Yang et al. (24)	N/A
**Small-cell lung cancer**	N/A	Arriola et al. (15) Antonia et al. (16) Reck et al. (17) Reck et al. (18)	N/A
**Renal cell carcinoma**	Yang et al. (34)	Hammers et al. (32) Motzer et al. (33)	N/A
**Urothelial carcinoma**	N/A	Galsky et al. (30) Sharma et al. (31)	N/A
**Pancreatic cancer**	Royal et al. (37)	Le et al. (36)	N/A
**Relapse of hematologic neoplasms after** **allo-HCT**	Davids et al. (41) Bashey et al. (42)	N/A	N/A
**Metastatic Sarcoma**	D’Angelo et al. (39)	N/A	N/A
**non-Hodgkin B-cell lymphoma**	Ansell et al. (40)	N/A	N/A
**gastric or gastroesophageal junction cancer**	Bang et al. (35)	N/A	N/A
**Pediatric patients with advanced solid tumors**	Merchant et al. (38)	N/A	N/A

While embracing the significance of what ipilimumab has brought to cancer immunotherapy, we should note that this immune-checkpoint inhibitor is also associated with side effects. The most common adverse effects related to ipilimumab are the immune-related ones (44). It is noteworthy that many studies on the effectiveness of ipilimumab were primarily designed in the context of malignant melanoma. Therefore, most of the immune-related adverse events (irAEs) discussed in this article occurred in the malignant melanoma setting. Still, some researchers have noted a similar or consistent safety profile in other cancers than melanoma (19, 27, 33, 35). Nevertheless, one study has proposed a favorable safety profile in unresectable locally advanced or metastatic Urothelial Carcinoma (31).

This similarity might help us predict AEs and correctly manage them in non-melanoma cancers, using the several findings reported in the melanoma settings. The findings related to AEs in non-melanoma settings are illustrated in **Table 2**. The studies observed an increase of 41% and 48% in total irAEs where ipilimumab was compared with placebo in monotherapy (26, 27) and 17% and 29% in the combination with chemotherapy (18, 19). However, the total treatment-related AEs were not substantially increased compared to irAEs, as 4/9 of the studies have reported an increase of less than 10% in this category for the ipilimumab subgroup (18, 19, 22, 31). In the two studies, one of the ipilimumab groups has even demonstrated a lower or equal rate of total AEs compared to placebo or no superior therapeutic effects in the combination therapy groups (22, 31).

**Table 2:** Summary of adverse events in non-melanoma cancers in clinical trials possessing ipilimumab group(s) of more than 50 patients and reporting safety profile.

ID	Study	Population for safety analysis	1. Mean/Median age 2. Female (%)	Target cancer	Dosage and concurrent induction therapies in Ipilimumab group	Incidence of total treatment-related AEs* (%)	Incidence of total irAEs (%)	Claiming a similarand/or consistent pattern to melanoma settings
**1**	(26)	1) n = 399 (Ipi) 2) n = 199 (placebo)	1. 1) 70 2) 69 2. 0% in both	mCRPC	4D of 10 mg/kg E3M	1) 82% 2) 49%	1) 77% 2) 29%	N/A^†^
**2**	(27)	1) n = 393 (Ipi) 2) n = 396 (placebo)	1. 1) 69 2) 67.5 2. 0% in both	mCRPC	4D of 10 mg/kg E3W	1) 75% 2) 45%	1) 63% 2) 22%	Yes
**3**	(19)	1) n = 388 (Ipi + chemo) 2) n = 361 (placebo + chemo)	1. 64 in both 2. 1) 16% 2) 14%	Advanced squamous NSCLC	4D of 10 mg/kg E3W + Paclitaxel and Carboplatin	1) 89% 2) 81%	1) 69% 2) 52%	Yes
**4**	(21)	1) n = 576 (Ipi + Nivol) 2) n = 391 (Nivol)	N/A (available for high tumor mutational burden subgroup)	Stage IV or recurrent NSCLC not previously treated with chemotherapy	4D of 1 mg/kg E6W + Nivolumab 3 mg/kg	1) 75.2% 2) 64.2%	N/A^†^	N/A^†^
**5**	(22)	1) n = 71 (concurrent Ipi) 2) n = 67 (phased Ipi) 3) n = 65 (placebo + chemo)	1. 1) 59 2) 61 3) 62 2. 1) 24% 2) 28% 3) 26%	Stage IIIB/IV or recurrent NSCLC	Concurrent Ipi: chemo + 4D of Ipi followed by 2D of placebo E3W Phased Ipi: chemo +4D of placebo followed by 2D of Ipi E3W	1) 76% 2) 82% 3) 80%	N/A^†^	N/A^†^
**6**	(16)	1) n = 54 (Ipi1 + Nivol3) 2) n = 98 (Nivol3) 3) n = 61 (Ipi3 + Nivol1)	1. 1) 61 2) 63 3) 66 2. 1) 22% 2) 37% 3) 26%	Recurrent SCLC	4D of 1 mg/kg Ipi E3W + Nivolumab 3 mg/kg	1) 74% 2) 53% 3) 79%	N/A^†^	N/A^†^
**7**	(18)	1) n = 478 (Ipi + chemo) 2) n = 476 (placebo + chemo)	1. 1) 62 2) 63 2. 1) 34% 2) 32%	Extensive-stage SCLC	4D of 10 mg/kg E3W + Etoposide and Platinum	1) 82% 2) 76%	1) 57% 2) 28%	N/A^†^
**8** ^a^	(33)	1) n = 547 (Ipi + Nivol) 2) n = 535 (Sunitinib)	1. both 62 2. 1) 25% 2) 28%	Advanced RCC	4D of 3 mg/kg E3W + Nivolumab 3 mg/kg	1) 93% 2) 97%	N/A^†^	Yes
**9**	(31)	1) n = 92 (Ipi3 + Nivol1) 2) n = 104 (Ipi1 + Nivol3) 3) n = 78 (Nivol3)	1. 1) 64.0 2) 63.0 3) 65.5 2. 1) 26% 2) 19% 3) 46%	Unresectable locally advanced or metastatic UC	1) 4D of 3 mg/kg E3W + Nivolumab 3 mg/kg 2) 4D of 1 mg/kg E3W + Nivolumab 3 mg/kg	1) 80.4% 2) 84.6% 3) 84.6%	N/A^†^	No (lower than melanoma settings)
**10**	(35)	1) n = 57 (Ipi) 2) n = 57 (Best supportive care)	1. 1) 65 2) 62 2. 1) 36.8% 2) 59%	Unresectable Locally Advanced/Metastatic Gastric or GEJ Cancer	4D of 10 mg/kg E3W	1) 71.9% 2) 55.6%	N/A^†^	Yes

*AE, Adverse events; irAE, Immune-related adverse events; mCRPC, metastatic Castration-Resistant Prostate Cancer; 4D, Four Doses; E3M, Every three months; E3W, Every three weeks; NSCLC, Non-small-cell lung cancer; SCLC, Small-cell lung cancer; RCC, Renal Cell Carcinoma; UC, Urothelial Carcinoma; GEJ, Gastroesophageal cancer. ^†^N/A, Not applicable due to no claims by the authors.

^a^This study does not compare ipilimumab to placebo.

Nivolumab has been the most promising immune-checkpoint inhibitor in recent years (45). Therefore, we have individually discussed the safety profiles of the studies reporting ipilimumab and nivolumab combination therapies (**Table 3**). Fatigue (16, 20, 31–33, 39, 46), pruritus (16, 20, 21, 31–33, 46), diarrhea (16, 20, 21, 31, 33, 46), rash (16, 21, 32, 46), loss of appetite (16, 32, 39), and nausea (20, 32) were the most common grade 1-2 (G1-2) treatment-related AEs in the ipilimumab and nivolumab combination therapies. Fortunately, most of these conditions did not turn into higher grade AEs and drug discontinuation. Diarrhea (16, 21, 31–33, 46), increased lipase (16, 20, 31–33), elevated alanine transaminase (ALT) (31, 32, 46), colitis (20, 31, 32), rash (16, 21), and anemia (21, 39) were commonly manifested among G3-4 AEs in patients received these monoclonal antibodies. Amylase was not significantly increased in the patients receiving ipilimumab. In fact, only three subgroups had an elevated amylase on their top 3 G3-4 AEs, all receiving nivolumab 3 mg/kg (alone, combined with ipilimumab 1 mg/kg, or 3 mg/kg) (31, 32). Neither of these studies has reported irAEs.

**Table 3:** Safety profile of the studies involving ipilimumab and nivolumab.

Study	Population for safety analysis	1. Mean/Median age 2. Female (%)	Target cancer	Incidence of total treatment-related AEs*	Incidence of G3-4 AEs	3 most common G1-2 AEs	3 most common G3-4 AEs
**(** 16 **)**	1) n = 54 (Ipi 1 mg/kg + Nivol 3 mg/kg) 2) n = 61 (Ipi 3 mg/kg + Nivol 1 mg/kg) 3) n = 98 (Nivol 3 mg/kg)	1. 1) 61 2) 66 3) 63 2. 1) 22% 2) 26% 3) 37%	Recurrent SCLC	1) 40 (74%) 2) 48 (79%) 3) 52 (53%)	1) 10 (19%) 2) 18 (30%) 3) 13 (13%)	1) 1. Fatigue: 12 (22%) 2. Diarrhea: 8 (15%) 3. Decreased appetite: 6 (11%) 2) 1. Fatigue: 16 (26%) 2. Pruritus: 11 (18%) 3. Diarrhea & Rash: 10 (16%) 3) 1. Pruritus: 11 (11%) 2. Fatigue: 10 (10%) 3. Diarrhea & Nausea: 7 (7%)	1) 1. Dyspnea: 2 (4%) 2. Several others: 1 (2%) 2) 1. Increased lipase: 5 (8%) 2. Diarrhea: 3 (5%) 3. Rash (maculopapular): 2 (3%) 3) 1. Several AEs: 1 (1%)
**(** 20 **)**	1) n = 39 (Ipi 1 mg/kg E6W + Nivol 3 mg/kg) 2) n = 38 (Ipi 1 mg/kg E12W + Nivol 3 mg/kg)	1. 1) 62 2) 68 2. 1) 38% 2) 55%	Recurrent stage IIIb or stage IV, chemotherapy-naive NSCLC	1) 28 (72%) 2) 31 (82%)	1) 13 (33%) 2) 14 (37%)	1) 1 & 2. Diarrhea & Fatigue: 8 (21%) 3. Pruritus & Nausea: 5 (13%) 2) 1. Pruritus: 9 (24%) 2. Diarrhea: 7 (18%) 3. Nausea: 6 (16%)	1) 1 & 2. Adrenal insufficiency & Colitis: 2 (5%) 3. Several others: 1 (3%) 2) 1. Increased lipase: 3 (8%) 2. Pneumonitis: 2 (5%) 3. Several others: 1 (3%)
**(** 21 **)^a^**	1) n = 576 (Ipi 1 mg/kg + Nivol 3 mg/kg) 2) n = 391 (Nivol 240 mg) 3) n = 570 Chemotherapy	N/A (available for high tumor mutational burden subgroup)	Stage IV or recurrent NSCLC not previously treated with chemotherapy	1) 433 (75.2%) 2) 251 (64.2%) 3) 460 (80.7%)	1) 180 (31.2%) 2) 74 (18.9%) 3) 206 (36.1%)	1) 1. Rash: 87 (15.1%) 2. Diarrhea: 85 (14.8%) 3. Pruritus: 78 (13.5%) 2) 1 & 2. Diarrhea & Fatigue: 41 (10.5%) 3. Rash: 40 (10.2%)	1) 1, 2 & 3. Anemia, Diarrhea, & Rash: 9 (1.6%) 2) 1 & 2. Diarrhea & Rash: 3 (0.8%) 3. Anemia, Asthenia, & Fatigue: 2 (0.5%)
**(** 31 **)**	1) n = 92 (Ipi 3 mg/kg + Nivol 1 mg/kg) 2) n = 104 (Ipi 1 mg/kg + Nivol 3 mg/kg) 3) n = 78 (Nivol 3 mg/kg)	1. 1) 64.0 2) 63.0 3) 65.5 2. 1) 26% 2) 19% 3) 46%	Platinum-pretreated unresectable locally advanced or metastatic UC	1) 74 (80.4%) 2) 88 (84.6%) 3) 66 (84.6%)	1) 36 (39.1%) 2) 32 (30.8%) 3) 21 (26.9%)	1) 1. Pruritus: 29 (31.5%) 2. Fatigue: 24 (26.1%) 3. Diarrhea: 21 (22.8%) 2) 1. Fatigue: 30 (28.8%) 2. Pruritus: 28 (26.9%) 3. Diarrhea: 19 (18.3%) 3) 1 & 2. Fatigue & Pruritus: 26 (33.3%) 3. Maculopapular Rash: 14 (17.9%)	1) 1. Diarrhea: 9 (9.8%) 2. Elevated ALT: 6 (6.5%) 3. Elevated lipase: 4 (4.3%) 2) 1 & 2. Elevated ALT & lipase: 6 (5.8%) 3. Diarrhea: 5 (4.8%) 3) 1. Elevated lipase: 5 (6.4%) 2. Elevated amylase: 4 (5.1%) 3. Maculopapular Rash: 3 (3.8%)
**(** 32 **)**	1) n = 6 (Ipi 3 mg/kg + Nivol 3 mg/kg) 2) n = 47 (Ipi 3 mg/kg + Nivol 1 mg/kg) 3) n = 47 (Ipi 1 mg/kg + Nivol 3 mg/kg)	1. 1) 54.8 2) 55.6 3) 53 2. 1) 16.7 2) 23.4 3) 8.5	Metastatic RCC	1) 6 (100%) 2) 45 (95.7%) 3) 43 (91.5%)	1) 5 (83.3%) 2) 29 (61.7%) 3) 18 (38.3%)	1) 1. Fatigue: 6 (100%) 2. Hypothyroidism: 5 (83.3%) 3. Arthralgia & Decreased appetite: 4 (66.7%) 2) 1. Fatigue: 29 (61.7%) 2. Nausea: 21 (44.7%) 3. Pruritus: 17 (36.2%) 3) 1. Fatigue: 24 (51.1%) 2 & 3. Pruritus & Rash: 15 (31.9%)	1) 1, 2, & 3. Headache, Increased amylase, & Increased lipase: 2 (33.3%) 2) 1. Increased lipase: 13 (27.7%) 2. Increased ALT: 10 (21.3%) 3. Diarrhea & Colitis: 7 (14.9%) 3) 1. Increased lipase: 7 (14.9%) 2 & 3. Diarrhea, Pyrexia, Increased ALT, AST, & amylase: 2 (4.3%)
**(** 33 **)^a^**	1) n = 547 (Ipi 3 mg/kg + Nivol 3 mg/kg) 2) n = 535 (Sunitinib)	1. both 62 2. 1) 25% 2) 28%	Previously untreated advanced RCC	1) 509 (93%) 2) 521 (97%)	1) 250 (46%) 2) 335 (63%)	1) 1. Fatigue: 179 (33%) 2. Pruritus: 151 (28%) 3. Diarrhea: 124 (23%)	1) 1. Increased lipase: 56 (10%) 2. Fatigue: 23 (4%) 3. Diarrhea: 21 (4%)
**(** 39 **)**	1) n = 42 (Ipi 1 mg/kg + Nivol 3 mg/kg) 2) n = 42 (Nivol 3 mg/kg)	1. 1) 54.1 2) 52.9 2. 1) 55% 2) 49%	Locally advanced, unresectable, or metastatic sarcoma	N/A^†^	N/A^†^	1) 1. Fatigue: 29 (69%) 2. Pain: 22 (52%) 3. Decreased appetite: 14 (33%) 2) 1. Fatigue: 24 (57%) 2. Pain: 23 (55%) 3. Cough: 13 (31%)	1) 1. Anemia: 8 (19%) 2 & 3. Hyponatremia & Hypotension: 4 (10%) 2) 1. Anemia: 4 (10%) 2. Decreased lymphocyte count: 3 (7%) 3. Several others: 2 (5%)
**(** 46 **)^b^**	1) n = 95 (Ipi 3 mg/kg + Nivol 1 mg/kg) 2) n = 47 (Ipi 3 mg/kg)	1. 1) 67 2) 52.9 2. 1) 34% 2) 32%	Metastatic melanoma not previously treated	1) 86 (91%) 2) 43 (93%)	1) 51 (54%) 2) 11 (24%)	1) 1. Rash: 39 (41%) 2. Diarrhea, Pruritus, & Fatigue: 32 (34%) 2) 1. Fatigue: 20 (43%) 2. Pruritus: 13 (28%) 3. Diarrhea & Rash: 12 (26%)	1) 1. Colitis: 16 (17%) 2 & 3. Diarrhea & Elevated ALT : 10 (11%) 2) 1. Diarrhea: 5 (11%) 2. Colitis: 3 (7%) 3. Hypophysitis: 2 (4%)

*AE, Adverse events; G3-4, Grade 3-4; SCLC, Small-cell lung cancer; E6W, Every six weeks; NSCLC, Non-small-cell lung cancer; UC, Urothelial Carcinoma; ALT, Alanine transaminase; RCC, Renal Cell Carcinoma; AST, Aspartate transaminase. ^†^N/A, Not applicable due to no claims by the authors.

^a^We did not enlist the most common AEs for the chemotherapy & sutinib subgroups to reduce unnecessary complications.

^b^This study is presented here for further comparison with the melanoma settings.

## Adverse Effects in Melanoma

In this section, we aim to cover the data published in clinical trials in the advanced melanoma setting. As our knowledge is rapidly increasing, a better understanding of the side effects of ipilimumab will help us to develop more comprehensive safe and effective approaches to manage these complications.

### Ipilimumab Monotherapy Versus Placebo

For two studies discussed in this part, the enrolled patients were at stage III melanoma and were eligible candidates for receiving 10 mg/kg adjuvant ipilimumab or placebo. Adjuvant ipilimumab or placebo was administered every three weeks for four doses, and then, every three months, for up to three years if a patient received complete treatment. In any case of consent withdrawal, disease progression, high toxicity, or death, the treatment discontinuation was implemented. Eggermont et al. (47) have reported that 465 of 471 (99%) of the patients in the ipilimumab arm, and 432 of 474 (91%) of the placebo arm had overall adverse events of any grades, with 254 (54%) and 118 (25%) G3-4 adverse events in each group, respectively. Version 3.0 of Common Terminology Criteria for Adverse Events (CTCAE) developed by the National Cancer Institute was utilized to report AEs. Total irAEs were more frequent in the ipilimumab arm than the placebo arm [426 (90.4%) vs. 183 (38.6%)]. The same findings were true for G3-4 irAEs [198 (42.0%) vs. 12 (2.5%)]. The most common G3-4 irAEs observed in the ipilimumab arm were gastrointestinal [75 (16%), 45 patients had diarrhea, and 36 had colitis], hepatic [50 (11%), half of the patients having increased liver function tests], and endocrine [40 (8%), 24 cases of hypophysitis and one case of hypothyroidism]. However, in the placebo arm, four patients manifested gastrointestinal complications, one patient hepatic irAEs and no-one demonstrated endocrine irAEs. Dermatological [277 (59%), pruritus in 176 and rash in 156 patients], gastrointestinal [142 (30%), mostly diarrhea and colitis], and endocrine [137 (29%), 62 cases of hypophysitis and 41 cases of hypothyroidism] were the most prevalent G1-2 irAEs in the ipilimumab group. Dermatological irAEs (277 G1-2 vs. 21 G3-4) and hypothyroidism (41 G1-2 vs. 1 G3-4) seemed to have fewer tendencies to severe cases. Drug-related deaths were five (1%) in ipilimumab 10 mg/kg and zero in the placebo group. Causes of the deaths related to treatment in the ipilimumab 10 mg/kg subgroup were colitis in three cases (two with gastrointestinal perforation), and myocarditis and multiorgan failure with Guillen-Barre syndrome each caused one death (47). A study conducted by Coens et al. (48) on 951 stage III cutaneous melanoma patients have demonstrated that ipilimumab could not lead to a clinically relevant decline in global health-related quality of life (HRQoL) compared with placebo.

### Ipilimumab Versus Placebo in Combination Therapies

Herein, we discuss irAEs data in the clinical trials investigating the ipilimumab versus placebo in combination therapies. Weber and colleagues have compared ipilimumab 3 mg/kg plus glycoprotein 100 melanoma antigen vaccine (gp100) (group A) and placebo plus gp100 (group B) (49). In this study, ipilimumab was administered up to four times every three weeks followed by the treatment continuation if the patients met the conditions. They found that overall treatment-related AEs, G3-4 AEs, overall irAEs, and G3-4 irAEs for group A (n = 131) were 105 (80.2%), 30 (22.9%), 80 (61.1%), and 19 (14.5%), respectively. For group B (n = 132) these numbers were 104 (78.8%), 15 (11.4%), 42 (31.8%), and 4 (3%). Deaths related to the treatment were four in group A and two for group B. Most of the adverse events, including G3-4 symptoms, were reversible using vigilant monitoring and treatment.

In another study (50), the patients randomized into ipilimumab 10 mg/kg plus dacarbazine (group A) and placebo plus dacarbazine (group B), given in four treatment cycles every three weeks. Then both groups were received dacarbazine alone every three weeks four times. Maintenance therapy using ipilimumab or placebo every 12 weeks was also recommended for candidates. Data of 247 patients from group A and 251 patients from group B were used for the safety assessment. All grades of AEs and irAEs were reported as 244 (98.8%) and 192 (77.7%) for group A and 236 (94%) and 96 (38.2%) for group B. G3-4 treatment-related AEs were more common among group A [139 (56.3%) versus 69 (27.5%) (P < 0.001)]. The number of patients with G3-4 irAEs was 103 (41.7%) against 15 (6.0%). No drug-related death was reported in group A, whereas one of the group B patients experienced death owing to gastrointestinal hemorrhage.

In another study, Robert et al. have demonstrated that seven patients who survived for at least five years following ipilimumab maintenance therapy have manifested grade 3 or 4 irAEs. Of five patients who were experienced irAEs of any grade, all had skin irAEs, two suffered from GI irAEs, two showed increased ALT or AST, and one had endocrine irAE. G3-4 irAEs were only observed in one patient and they were restricted to the skin (51).

The safety profile of the studies discussed in this group resembles the ones in the previous section. Although the total AEs remained similar, G3-4 AEs and irAEs were increased in the ipilimumab subgroup. Therefore, G3-4 irAEs surged to the highest level where ipilimumab was added to the treatment regimen. Furthermore, no study in both sections was reported a significant increase in the mortality rate of the patients receiving ipilimumab.

### Ipilimumab Dose Comparison

Understanding and comparing the side effects of various doses of ipilimumab and will help the patients suffer less where higher AEs overshadow the clinical benefit. Ascierto and colleagues (52) have demonstrated that the overall adverse events and irAEs with any grade were 286/364 (79%) and 269/364 (74%) of the patients in 10 mg/kg group, and 228/362 (63%) and 197/362 (54%) in 3 mg/kg group, respectively. G3-4 irAEs were also seen in 110 (30%) of the 10 mg/kg subgroup and 50 (14%) of the 3 mg/kg arm. Diarrhea [10 mg/kg arm: 37 (10%) vs. 3 mg/kg arm: 21 (6%)], colitis [10 mg/kg arm: 19 (5%) vs. 3 mg/kg arm: 9 (2%)], and increased alanine aminotransferase [10 mg/kg arm: 12 (3%) vs. 3 mg/kg arm: 2 (1%)] were accounted for the most common G3-4 treatment-related AEs. Death due to irAEs was noted in a patient in 3 mg/kg group owing to large intestinal perforation. In another study, overall irAEs were closer for 10 mg/kg [50 of 71 (70.4%)] and 3 mg/kg [46 of 71 (64.8%)], where compared with 0.3 mg/kg arm [19 of 72 (26.4%)]. No patient was developed G3-4 irAEs in the 0.3 mg/kg arm. However, five patients in the 3 mg/kg subgroup and 18 in the 10 mg/kg arm were diagnosed with G3-4 irAEs. The most common observed irAEs were gastrointestinal (11 in the 10 mg/kg subgroup, two in the 3 mg/kg) (53). Following a survey on patients developing hypophysitis, Albarel et al. (54) have found that although 62 of the total 131 patients were received ipilimumab at a 3 mg/kg dosage, 11/15 of the patients developing this condition were treated at a dosage of 10 mg/kg. Interestingly, all 15 patients have suffered from at least one hormonal deficiency. While the number of tyrotroph, gonadotroph, and corticotroph deficiencies was similar (13, 12, and 11, respectively), no antidiuretic hormone (ADH) deficiency (diabetes insipidus) was observed. This finding points out that ADH is probably not affected or much less negatively affected by ipilimumab therapy. Clinical symptoms were usually improved swiftly on high-dose glucocorticoids or by physiological replacement doses. Apart from corticotroph deficiency cases, hormonal deficiencies were also improved. In a study (55), ipilimumab was administered every three weeks to advanced melanoma adolescent patients aging 12–18. They have reported that one out of four patients receiving 3 mg/kg ipilimumab treatment was developed G3-4 irAEs, compared to 5 out of 8 patients receiving 10 mg/kg ipilimumab.

Overall, healthcare providers should be aware that patients treated with ipilimumab might develop irAEs. It is demonstrated that most irAEs can be reversible (56). Specific practical guidelines have been developed to help patients and practitioners to manage irAEs (57, 58). Systemic corticosteroids might be required in 35% of the patients, and 10% may further need anti-TNFα for immune-suppression. These treatments could not alter overall survival or time to treatment failure (14). Endocrinopathies such as hypophysitis, although not frequent, might be too severe and practitioners may, therefore, seek long-term hormone replacement therapy (57).

Altogether, the overall evidence suggests that the safety profile might be independent or slightly dependent on underlying cancer (**Tables 2** and **3**). However, some studies may confront this claim and show variations related to different tumor types (31). For example, G3-4 hypophysitis might be more pronounced in melanoma settings (46, 47, 54). Concurrently, a study on the patients with advanced or metastatic sarcoma receiving ipilimumab plus nivolumab (39) has shown a difference in other settings in which the patients received the same treatment (**Table 3**). Nevertheless, the lack of enough information on some conditions in the studies or random incidents might have resulted in these variations. Therefore, careful meta-analyses need to address this matter.

The pattern of AEs seems to be related more with the type of therapy (i.e., the dosage of ipilimumab and the type of concurrent therapies) (39, 50). Increasing the dosage may impose a higher risk of AEs while providing an overall advantage. Furthermore, the administration of other agents may increase the risk of specific types of AEs. An example is an excessive increase in alanine and aspartate aminotransferase (ALT and AST) where ipilimumab is co-administered with dacarbazine, a known hepatotoxic agent, in the metastatic melanoma settings (50). We suggest that evaluation of AEs should be conducted in every clinical trial in particular in the context of combination therapies.

## Conclusion

Ipilimumab has shown promising results in many forms of advanced cancers evidenced by numerous trials. However, clinicians should always bear in mind that these benefits come at the cost of adverse events. Patients should always be informed about these side effects because their awareness might influence their trust and hope. Conversely, no awareness might result in their perplexity. We know that if the patients lose their trust in their treatment and attending physicians, they might lose their last chance of getting cured. Furthermore, when the signs and symptoms of adverse events start to manifest, timely management should be executed according to the approved guidelines and protocols. Further studies may require deciphering the hidden aspects of this area.

## Author Contributions 

Conception and design of the paper were done by AK and HM. The initial draft was completed by AK. Editing and further drafts were done by HM. SA contributed to the critical discussion, visual design, and figure illustration. All authors contributed to the article and approved the submitted version.

## Conflict of Interest

The authors declare that the research was conducted in the absence of any commercial or financial relationships that could be construed as a potential conflict of interest.
